# Machine Learning applied to student attentiveness detection: Using emotional and non-emotional measures

**DOI:** 10.1007/s10639-023-11814-5

**Published:** 2023-05-01

**Authors:** Mohamed Elbawab, Roberto Henriques

**Affiliations:** NOVA Information Management School (NOVA IMS), Universidade Nova de Lisboa, 1070-312 Lisbon, Portugal

**Keywords:** Machine Learning, E-learning, Learning Analytics, Extreme gradient boosting, Accuracy, AUROC

## Abstract

Electronic learning (e-learning) is considered the new norm of learning. One of the significant drawbacks of e-learning in comparison to the traditional classroom is that teachers cannot monitor the students' attentiveness. Previous literature used physical facial features or emotional states in detecting attentiveness. Other studies proposed combining physical and emotional facial features; however, a mixed model that only used a webcam was not tested. The study objective is to develop a machine learning (ML) model that automatically estimates students' attentiveness during e-learning classes using only a webcam. The model would help in evaluating teaching methods for e-learning. This study collected videos from seven students. The webcam of personal computers is used to obtain a video, from which we build a feature set that characterizes a student's physical and emotional state based on their face. This characterization includes eye aspect ratio (EAR), Yawn aspect ratio (YAR), head pose, and emotional states.

A total of eleven variables are used in the training and validation of the model. ML algorithms are used to estimate individual students' attention levels. The ML models tested are decision trees, random forests, support vector machines (SVM), and extreme gradient boosting (XGBoost). Human observers' estimation of attention level is used as a reference. Our best attention classifier is the XGBoost, which achieved an average accuracy of 80.52%, with an AUROC OVR of 92.12%. The results indicate that a combination of emotional and non-emotional measures can generate a classifier with an accuracy comparable to other attentiveness studies. The study would also help assess the e-learning lectures through students' attentiveness. Hence will assist in developing the e-learning lectures by generating an attentiveness report for the tested lecture.

## Introduction 

Electronic learning (e-learning) is essential in the current educational system (Maatuk et al., 2022). E-learning went from an option to a necessity with the Covid-19 pandemic (Mellieon & Robinson, 2021). E-learning can be performed using laptops, desktops, tablets, or other devices, while the students are not physically located in the university/school (Mellieon & Robinson, 2021). E-learning has spread rapidly due to its provision of learning in different formats (Deng & Wu 2018). On the other hand, the teachers could not classify students' behavior or monitor them while delivering the lectures remotely (Shah et al., 2021). Subsequently, teachers need the aid of artificial intelligence (AI), new technological innovations, advancements, and computers to develop education (Alam, 2021; Chen et al., 2020).

Artificial Intelligence (AI) has impacted education by improving efficiency, personalized learning, more thoughtful content, and effectiveness, thereby improving the learning experience and overall quality of learning (Alam, 2021; Chen et al., 2020; Romero & Ventura, 2010). AI is increasingly used in education in various forms, including automation of administrative processes and tasks, curriculum and content development, and instructions and learning processes (Chen et al., 2020; Mellieon & Robinson, 2021). Moreover, AI in education has applications in three main categories: Administration, Instruction, and Learning (Alam, 2021; Chen et al., 2020).

The administration category focuses on administrative functions such as grading exams, providing students' feedback, identifying students' learning styles and preferences, and assisting instructors in data-driven work (Alam, 2021; Chen et al., 2020; Hwang et al., 2020).

The instruction category focuses on teaching functions like tailoring teaching methods based on the students, analyzing the syllabus and course materials, predicting student dropout, and using virtual reality to provide practical experience to students (Alam, 2021; Chen et al., 2020; Gligorić et al., 2012).

As for the learning category, it leverages an integral part of education: learning (Alam, 2021; Chen et al., 2020; Kučak et al., 2018). Examples of the learning category include customizing the university course selection for students, detecting students' learning states, and uncovering students' learning needs and shortcomings so they can be addressed on time (Alam, 2021; Chen et al., 2020).

From a technical perspective, AI in education can be implemented using machine learning (ML), learning analytics, and data mining (Alam, 2021; Chen et al., 2020; Romero & Ventura, 2010). The core of ML is knowledge discovery through generating meaningful patterns (Chen et al., 2020). ML can help students when choosing a university. The ML model can recommend the best-fit university for each student (Chen et al., 2020). ML can also help to generate computerized adaptive assessments for students (Romero & Ventura, 2010). Using text mining, ML can also help analyze students' handwritten assessment papers (Chen et al., 2020; Romero & Ventura, 2010). Learning analytics in education introduces the new technology of ML, data visualization, learning sciences, and semantics applied to education (Chen et al., 2020). Teachers can use learning analytics to understand the degree of understanding of the students of different concepts; subsequently, the teacher can adjust the teaching method (Chen et al., 2020). Learning analytics can also detect dropout rates, which would help the school to improve retention rates (Chen et al., 2020; Romero & Ventura, 2010). Educational data mining utilizes ML and data mining algorithms over educational data to solve issues in the field of education (Matzavela & Alepis, 2021; Chen et al., 2020; Bakhshinategh et al., 2018; Negron & Graves, 2017). E-learning generates a large amount of data that could be used in educational data mining (Negron & Graves, 2017). Examples of educational data mining include 1) predicting student performance based on existing data; and 2) a better understanding of the learning process leading to a better understanding of the educational setting (Chen et al., 2020; Negron & Graves, 2017).

The three mentioned technical implementations of AI in Education (ML, learning analytics, and educational data mining) are firmly related, where two communities evolved: learning analytics and educational data mining (Chen et al., 2020). Both communities share the same interest in using a data-intensive approach, share the techniques and finally share the same goal of enhancing educational practices (Chen et al., 2020; Romero & Ventura, 2020). However, the difference between learning analytics and educational data mining is in focus; learning analytics focuses on the educational challenge, while educational data mining focuses on the technological challenge (Romero & Ventura, 2020). To furtherly elaborate on the differences, data analytics focuses on data-driven decision-making and using predictive models in the different dimensions of learning. In contrast, educational data mining focuses on looking for new data patterns and developing new algorithms and models (Romero & Ventura, 2020).

Several issues have emerged with e-learning; the three main categories that have emerged are connectivity (e.g., the disruption of the teaching affected by unstable internet), teaching technology (e.g., the platform of learning being not satisfactory), and interactivity (e.g., the decrease of the focus of students) (Karjo et al., 2022; Yusuf & Ahmad, 2020). Another significant issue in e-learning is that the teachers cannot monitor the students and classify their behavior as in a traditional classroom (Shah et al., 2021). Hence the idea of using AI to monitor students has emerged. In the last decade, student behavior has caught the attention of researchers in computer vision and e-learning (Jalal & Mahmood, 2019). For monitoring the students, two concepts have emerged. These are student engagement and attentiveness (Saini & Goel, 2019, Negron & Graves, 2017). Student attentiveness measures the student being on a task, while engagement goes beyond that (Saini & Goel, 2019). Student engagement measures the student being on a task while mentally and emotionally invested in the activity (Saini & Goel, 2019). Engagement is hard to be measured by physical means, as what distinguishes engagement from attentiveness is the internal thought or feeling (Saini & Goel, 2019, Negron & Graves, 2017). Consequently, we will focus on measuring students' attentiveness in this study.

This study aims to estimate individual students' attention levels using a video taken from students. We propose using a combination of emotional and non-emotional measures extracted from those videos, allowing us to model accurate students' attentiveness classifiers.

The remainder of this paper is organized as follows. Section 2 contains a review of the relevant literature. Section 3 describes the methods. Our experimental setup is described in Sect. 4. Section 5 presents the numerical results and discussion. The last section is devoted to the conclusions, limitations, and recommendations for future work.

## Literature review 

One of the biggest challenges for the education system in the current period is the outbreak of the Covid-19 pandemic (Gherhes et al., 2021). At least 1.6 billion people were affected by the closure of schools in more than 190 countries due to the possible transmission of Covid-19 (Gherhes et al., 2021). Hence, face-to-face learning shifted to e-learning, where teachers and students had to adapt their behaviors, teaching styles, learning styles, and assessment methods.

Student attentiveness is one of the biggest challenges in the classroom experience (Zaletelj & Košir, 2017) but remains a constant topic for discussion in cognitive psychology (Shah et al., 2021). Attentiveness is described as the sustained focus of cognitive resources on information while ignoring distractions Deng & Wu 2018). Sustained attentiveness during classes is essential to learning success (Deng & Wu 2018). The problem with online classes is that students are less attentive and more distracted in a more favorable environment, usually their home (Shah et al., 2021).

Student attentiveness studies started developing in smart classrooms, where multiple sensors collect the data (Zaletelj & Košir, 2017). The study of (Gligorić et al., 2012) aims to provide real-time automatic feedback based on student fidgeting and student noise. Gligorić et al.'s (2012) study used passive infrared (PIR) motion sensors, microphones, video cameras, and sound sensors. Zaletelj & Košir study used a Microsoft Kinect sensor to collect data in a study developed at a public university in Slovenia (Zaletelj & Košir, 2017). Zaletelj & Košir tested 7 ML methods from simple models to more complex ones. Examples of the models are simple decision trees and weighted *k* nearest neighbor (k-NN). The authors used different features from the tested participants, including sitting position, eye and mouth openness, and gaze point. The study achieved an average accuracy of 75.3%.

Tabassum et al. (2020) also assessed attentiveness in a classroom without special sensors, as only a camera was used. In the study, students' facial expressions were used to identify their attentiveness using neural networks. Videos of participants were captured while attending a classroom, and then these videos were processed through a motion detection algorithm that captured images of students from the videos. These students' images went through the Amazon Rekognition system to produce the set of expression analyses for each face image. These expressions were used among the labeled data to train the system. The model achieved an accuracy of 93.14% using only the students' emotions.

Other studies assessing student attentiveness were developed in e-learning settings. The study Chen developed in 2012 assessed learners' attention to e-learning based on three aspects extracted from facial features. The features used in Chen's study are avoidance (frequency of facial movement), concentration (eye movement), and happiness (the distance between the lips). The higher the sum of these scores, the more attention is indicated. The study did not use ML techniques for prediction; however, the study explored the relationship between facial recognition and the process of learning. The study's main conclusion is that recognizing students' facial expressions can be used to understand the level of attention.

In the study by Deng & Wu, the authors detected the face and then the eyes. The authors propose an attention score based on the detected face and eyes. This score is calculated by summing three values representing face detection, eye detection, and eye openness. The eye openness is based on an eye state classifier that uses ML(Deng & Wu 2018). The best classification model has achieved an accuracy of 93.1%.

Revadekar et al. (2020) proposed three independent models of measuring attention. Revadekar et al.'s study models are posture-based attention detection, emotion-based attention detection, and drowsiness detection. However, only posture-based attention detection was tested in the study. The authors identified five postures: attentive, head resting on the hand, leaning back, writing, and not looking at the screen. The posture model achieved an accuracy of 99.82%.

In (Shah et al., 2021), the authors proposed a combined model using head pose estimation, emotional classification, and drowsiness detection for classifying the student's learning level. This model was a theoretical hypothesis since the authors did not test this combined model. Table 1 accommodates some of the key pros and cons of the previous literature that used artificial intelligence in detecting student attentiveness.

**Table 1:** Pros and Cons of most relevant work developed on literature student attentiveness detection

Reference	Pros	Cons	Accuracy
Gligorić et al., 2012	An innovative approach to the smart classroom where a combination of sound and movement existence used as inputs	Need specific types of sensors	NA
Chen, 2012	Used multiple ways to measure attentiveness	Measured only one emotion (happiness) Measured happiness from the distance between lips Not using ML	NA
Zaletelj & Košir, 2017	Seven ML methods tested	Need Kinect sensor	75.3%
Deng & Wu 2018	Three classifiers tested	Used only the eye state to detect attentiveness level	93.1%
Tabassum et al., 2020	Amazon Rekognition for extracting emotions	Only used emotions	93.14%
Revadekar et al., 2020	Three independent models proposed	One model tested Did not combine models No classifiers mentioned	99.82%
Shah et al., 2021	Combine multiple detection methods	Model not tested	NA

As the studies shown in Table 1, no model containing emotional and non-emotional features has been tested while only using a webcam. Gherheș et al. (2021) have called for the necessity to model students' behaviors in e-learning. Therefore in this study, we employ and test an ML model that measures student attentiveness based on combining drowsiness detection, head position, and emotion detection. The novelty of the work is as follows:

Testing the combined model of emotional and non-emotional measures in one system

Building a combined model on top of a verified emotion detection model

The new results help in understanding students' behaviors in e-learning

## Methodology

**Fig. 1:**
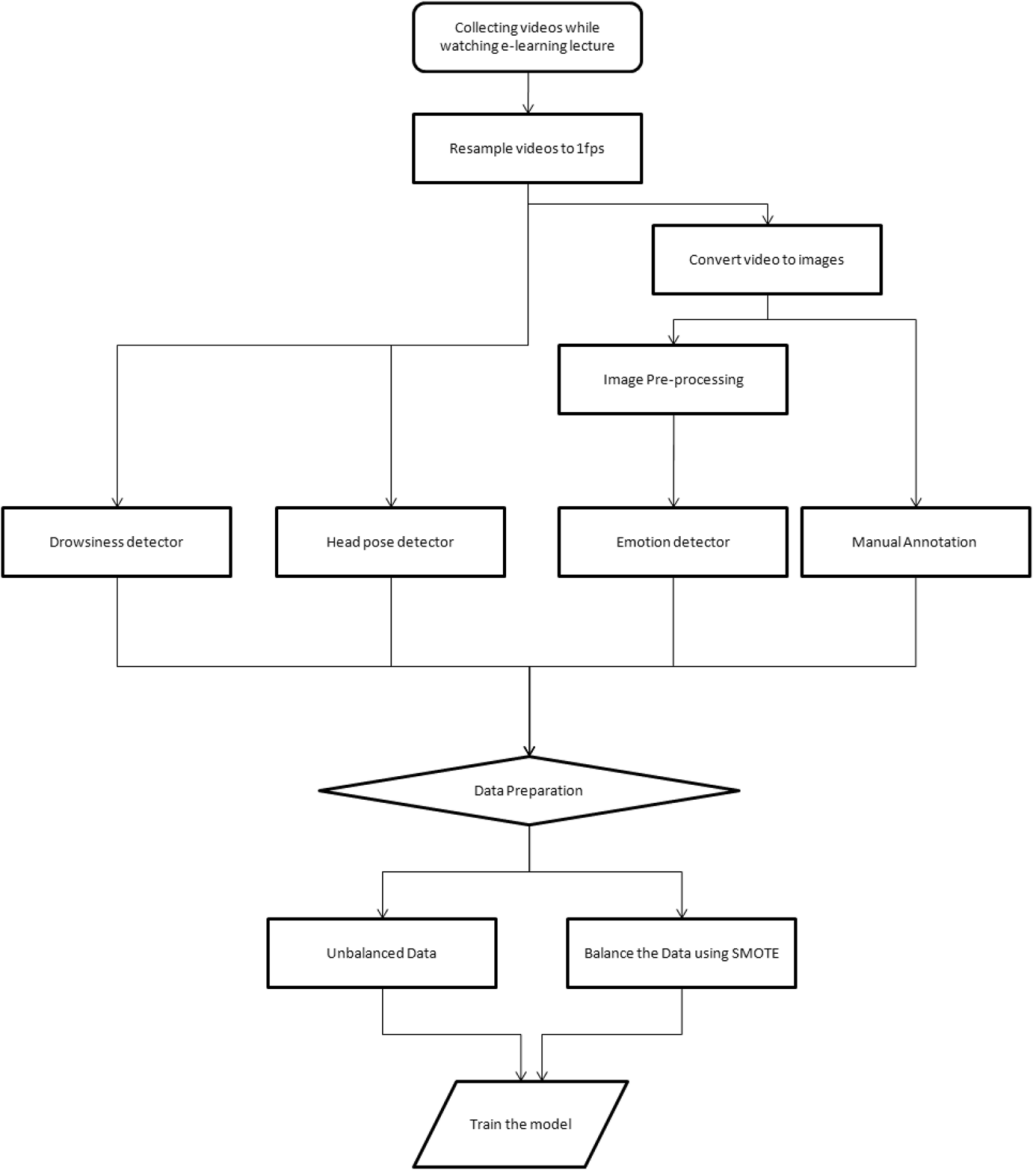
Methodology flow chart

This section presents the methods, data preparation, machine learning algorithms and their performing metrics. As mentioned in the previous section, we will develop and test a model that measures student attentiveness by combining drowsiness detection, head position, and emotion detection. First, the student-recorded video is processed by the drowsiness and head pose detectors. In parallel, the video is converted into images representing one frame per 1 s. The emotion detector will then process these images. Manual annotation is also processed on these images to detect the attentiveness level of the student. Figure 1 presents the methodology steps.

### Drowsiness detector

**Fig. 2:**
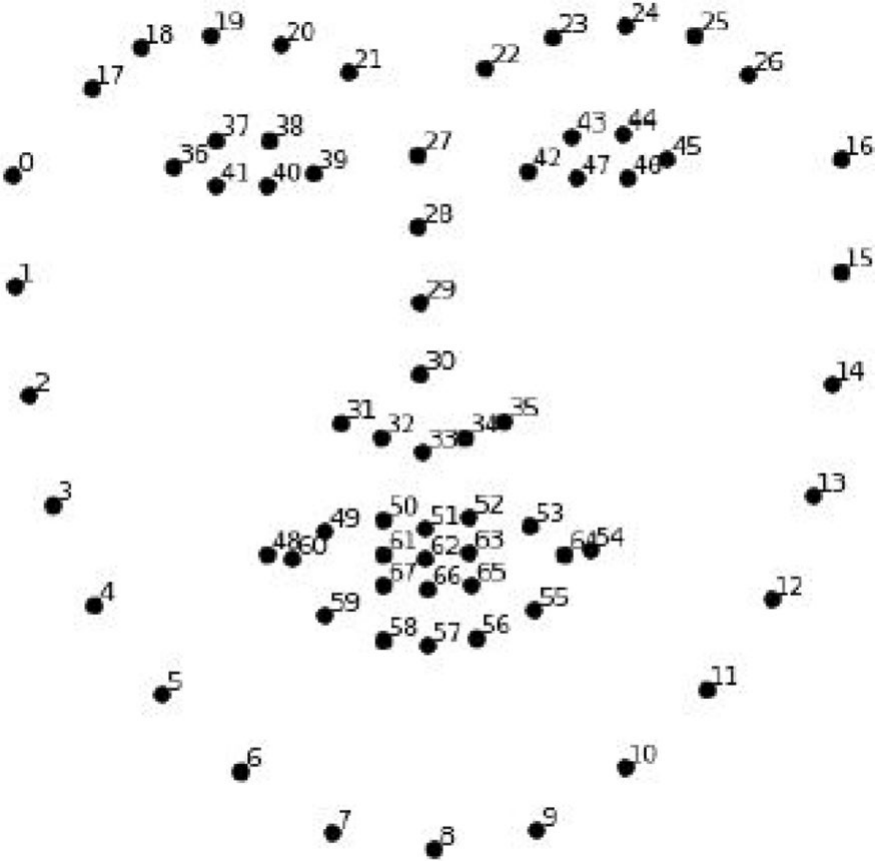
Presentation of the 68 Facial landmarks (Korshunov & Marcel, 2018)

The first step in the drowsiness detector is detecting the face, where a 68-point structure is distributed among the recognized key points in a human face. Then the drowsiness of the student will be calculated using the eye aspect ratio (EAR) and the yawn aspect ratio (YAR) (Shah et al., 2021). Figure 2 shows the presentation of the 68 facial landmarks (Pinzon-Gonzalez & Barba-Guaman, 2022).

The EAR is defined as (Shah et al., 2021)

1 $$EAR=\frac{EAR 1+EAR 2}{2}$$

2 $$EAR 1=\frac{\left|\left|37-41\right|\right|+\left|\left|38-40\right|\right|}{2 \left|\left|36-39\right|\right|}$$

3 $$EAR 2=\frac{\left|\left|43-47\right|\right|+\left|\left|44-46\right|\right|}{2 \left|\left|42-45\right|\right|}$$

The YAR is defined as (Shah et al., 2021)

4 $$YAR=\frac{\left|\left|61-67\right|\right|+\left|\left|62-66\right|\right|+\left|\left|63-65\right|\right|}{\left|\left|64-60\right|\right|}$$

EAR and YAR are calculated by Eq. 1 and Eq. 4 for drowsiness detection. The videos will be processed using the OpenCV library (Howse, 2013). For each frame, the EAR and the YAR are extracted. Hence the EAR and YAR are calculated for each second for each student. A threshold for the EAR indicates when the eyes are closed, indicated as sleeping (Shah et al., 2021). A threshold for YAR indicates when the mouth is opened widely, indicated as yawning (Shah et al., 2021). The drowsiness detector generates the first two inputs that we have for our ML model.

### Head pose detector

The head pose can help show the student's distraction or attentiveness (Pinzon-Gonzalez & Barba-Guaman, 2022). When a student is distracted, they may start looking here and there (Shah et al., 2021). Hence, the head's position may help recognize students' attentiveness and assist in training the ML model. First, a face mesh is built to identify the face and its six key points. Then the rotation angle is calculated. The X and Y components of the rotation angle are determined using the OpenCV library (Howse, 2013). For each frame, the two components of the rotation angle are extracted. Hence the two components of the rotation angle are calculated for each second for each student generating two more inputs for our ML model.

### Emotions' detector

Several authors have requested combining emotions with other measurement forms to assess student attentiveness (Revadekar et al., 2020; Shah et al., 2021), as facial expressions are one of the most potent signals for human beings to transfer their emotional states (Li & Deng, 2020). Facial expression recognition (FER) has been used in various study types, including driver fatigue surveillance, student attentiveness, and medical treatment (Khaireddin & Chen, 2021; Li & Deng, 2020). FER has been used to encode expression representation from facial representations. One of the famously used datasets for FER is FER 2013(Goodfellow et al., 2013; Khaireddin & Chen, 2021; Li & Deng, 2020). FER2013 is considered a benchmark in comparing performance for emotion recognition (Khaireddin & Chen, 2021). In (Khaireddin & Chen, 2021), the authors used convolution neural networks (CNN) where they adopted VGGNet architecture. Khaireddin & Chen fine-tuned the hyperparameters and experimented with various optimization methods for the VGGNet, where their model achieved an accuracy of 73.28% on FER2013 without extra training data. The VGGNet consists of four convolutional stages and three fully connected layers.

In this study, the emotion detector will implement the model developed and trained (Khaireddin & Chen, 2021) based on the FER2013 dataset. The re-sampled videos are split into pictures representing one frame per second. The images of each student representing each frame will first be preprocessed to fit the model proposed in (Khaireddin & Chen, 2021). The preprocessing starts with extracting the face from the image using OpenCV and the cascade file. The cascade file helps generate a cropped image containing only the face of the student. Then these generated images are grey-scaled. Then the images are scaled to 40 × 40 pixels and normalized as per the pre-trained model variables. To sum up, the preprocessing steps are:

1. The student's face is extracted from each image using OpenCV and the cascade file.
2. Each image is grey scaled
3. Each image is scaled to 40 × 40 pixels
4. Each image is standardized (dividing each pixel by 255)

Finally, using a VGGNet variant proposed by (Khaireddin & Chen, 2021), the emotion detector generates seven numerical variables for each frame, representing anger, disgust, fear, happiness, sadness, surprise, and neutral emotions. The emotion detector generates the last seven inputs used in the ML model, each representing the respective student's emotion for each second.

### Machine learning algorithms

In this proposal, we applied four different ML algorithms to the comparison, ranging in flexibility from simple models like decision trees to more complex models. The four ML algorithms are decision trees (Zaletelj & Košir, 2017), random forest (Yan 2021), SVM (Deng & Wu 2018), and XGBoost (Yan 2021).

#### Decision trees

A decision tree is one of the most popular supervised ML techniques that help in classification problems. A decision tree is easy to understand and interpret, hence used as our first model. Decision trees can be implemented in educational predictive models. Decision trees are constructed through an approach of an algorithm where it identifies ways to split the data based on conditions (Matzavela & Alepis, 2021). These conditions are generally in the form of an if–then-else statement. The deeper the tree, the fitter the model is. A classification decision tree can always be expressed as a tree-like graph with nodes, edges, and leaves. The nodes represent the question, the edges represent the answer, and the leaves correspond to the target classification (Matzavela & Alepis, 2021).

#### Random Forest

Random Forest is an extension of the bagging idea, where it can be used in classification problems. Random forests have many advantages, including being relatively fast to train, easily implemented in parallel, handling regression and classification, and being used for high-dimensional problems (Cutler et al., 2012). A random forest is a tree-based ensemble where each tree depends on a collection of random variables. Subsequently, it is used as the second model after the decision trees. The more decision trees are used in the random forest, the better prediction of the model (Cutler et al., 2012).

#### Support vector machines

Support vector machines (SVM) are one of the ML classification approaches. Many advantages are identified for the SVM, including reaching a global solution, good solution generalization properties, and clear geometric intuition on the classification task (Mavroforakis & Theodoridis, 2006). SVM constructs a hyperplane, or multiple hyperplanes, which can be used for classification or regression. A good separation is achieved by the hyperplane that provides the largest distance to the nearest training-data point of any class (Deng & Wu 2018). Hence, this method is the third to be tested within this study.

#### Extreme gradient boosting

Extreme gradient boosting (XGBoost) is an efficient and scalable implementation of the gradient boosting framework. Gradient boosting is an algorithm where new models are created that predict the residuals of prior models, and then these predictions are added together to make the final prediction. Gradient boosting uses a gradient descent algorithm to minimize the loss when adding new models. XGBoost is a cutting-edge application of gradient boosting machines where it has proven to push the limits of computing power for boosted trees algorithms. It is developed to increase the model performance with faster speed. Boosting is an ensemble technique in which new models are added to adjust the errors made by existing models. In XGB, Models are added recursively until no noticeable improvements are detected (Chen et al., 2016).

### Performance metrics for model evaluation

As per the studies that assessed students' attention (Chen, 2012; Deng & Wu 2018; Revadekar et al., 2020; Tabassum et al., 2020; Zaletelj & Košir, 2017), the main focus has always been on the accuracy of the model. We also used the area under the ROC curve (AUROC), as it summarizes a classifier's precision and recall in one value.

#### Accuracy

The confusion matrix and its underlying values are used to visualize the performance of supervised learning, where the True positive (Tp), True negative (Tn), false positive (Fp), and false negative (Fn) are used. The values are described as Tp: correct positive prediction, Tn: correct negative prediction, Fp: incorrect positive prediction, and Fn: incorrect negative prediction (Hasan et al., 2020). Accuracy is defined as per (Hasan et al., 2020)

5 $$Accuracy= \frac{Tp+Tn}{Tp+Tn+Fp+Fn}$$

#### AUROC

The area under the receiver operating characteristic (*AUROC) is* a performance metric used to evaluate classification models. The higher the AUROC, the better the model predicts the correct class of each observation. The AUROC was initially used for binary classification; hence AUROC one versus rest (OVR) will be used as it extends the use of AUROC to evaluate multi-classification problems. OVR computes the AUC of each class against the rest (Domingos & Provost, 2000; Fawcett, 2006). The OVR is Sensitive to class imbalance; hence it is used to show the impact of using and not using oversampling.

## Experimental setup

The goal of this experiment is to record student behavior while attending e-learning classes. A survey was developed and shared by email to students for the study. The survey included the purpose of the study, the video lecture to be watched, and instructions for recording a video of themselves while watching the lecture video. The participants were five females and three males from different university programs, including master's, postgraduate, and Ph.D. students. The lecture video topic is "Data Analysis Clustering and Classification". The lecture interested all participants as they are either students at the information management school or are researching similar topics. After recording the video, students were also asked to respond to a follow-up survey. This follow-up survey is an adapted subscale of attending behaviors from (Ford et al., 2000), where the responses were collected on a five-point Likert-type scale. The Likert-type scale ranged from 1 (strongly disagree) to 5 (strongly agree). The recordings were performed by students in their houses or preferred places, as with any e-learning class. The students were requested to use a built-in webcam that would be directed to their faces while recording. The lecture duration is 26 min and 58 s. This lecture was chosen because its duration is longer than 10–15 min; existing research claims that the student concentration drops after 10–15 min into lectures (Lim, 2017). Consequently, a video lecture longer than 15 min captures students' behavior changes.

### Data collection

Eight students responded by sharing their videos and answering the follow-up survey. One video was disregarded as it was not recorded correctly, leaving seven videos for development and analysis. The received videos ranged from 14 to 30 frames/ second. This study's videos were re-sampled into one frame per second (Zaletelj & Košir, 2017), as we would analyze the student once each second. This resulted in about 11,300 samples.

### Data annotation

In the previous literature, the definition of students' attentiveness in a class is scarce (Zaletelj & Košir, 2017). Hence we have followed the approach of (Zaletelj & Košir, 2017), where human observers analyzed the video recordings and annotated each of the frames for all students. The annotators were asked to record how attentive students were at each recording frame on a scale of 1, 3, and 5. One indicates not attentive, three indicates moderately attentive, and five indicates highly attentive. We have used the guidelines of (Goldberg et al., 2021) in developing the annotation. An example of being not attentive is being distracted by answering a phone call, while an example of being moderately attentive is when the gaze is shifting away, and finally, an example of being highly attentive is being in an upright position, listening, and looking to the screen. The first annotator is a developer of this study and is a master's degree student in data science. The second annotator is an expert in learning with ten years of experience teaching students at a university level. Both annotators have evaluated the frames. If the scoring matches, then it is accepted. However, if the scoring between the two annotators differed, then the output is revised by the expert that gives the final output.

### Data preparation

Eleven variables are input into the machine learning model to predict students' attentiveness. The variables are EAR, YAR, the x component of the rotation angle, the y component of the rotation angle, anger, disgust, fear, happiness, sadness, surprise, and neutrality.

While extracting the face from images on the emotion detector model, some faces were not detected; consequently, we did not detect any emotions. These frames were then removed, and this cleaning step resulted in a total of 10,899 frames being processed.

We used 80% of the data as a training set and 20% as a testing set to build the classifiers. We then used a tenfold cross-validation technique on the training set to avoid model overfitting and for hyperparameter tuning. The dataset is randomly divided into ten equal folds, each with approximately the same number of records; 10 validation experiments are then performed, each used in turn as the validation set and the remaining nine used as the training set. We then used the 20% testing set to evaluate the model performance (Berrar, 2018).

**Fig. 3:**
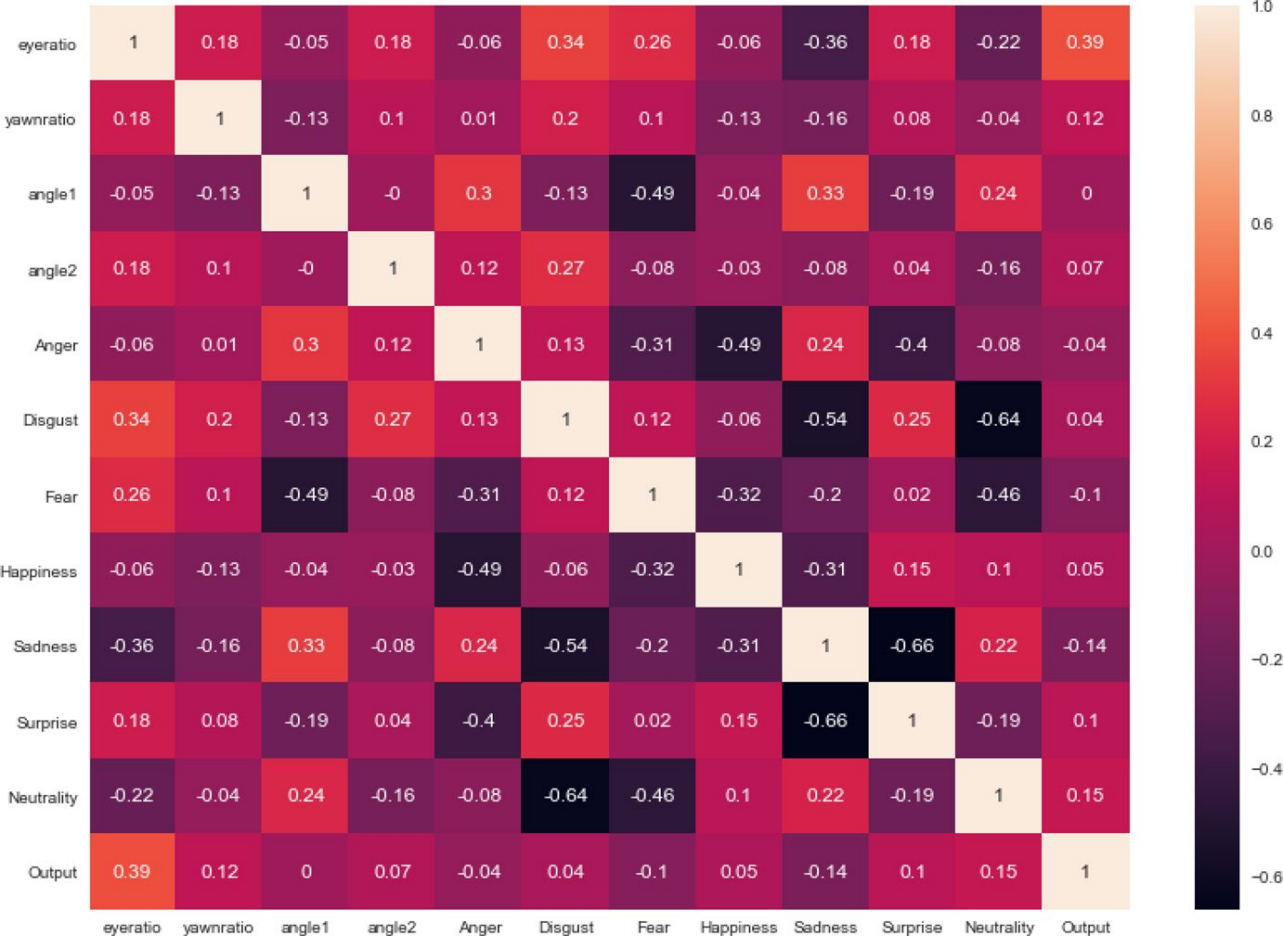
Heatmap for the input variables and the output

Before training the predictive models using the 11 input variables, we built a heatmap with the Pearson correlations among features. As shown in Fig. 3, the only noticeable correlation was between disgust and neutrality. However, we did not remove any of the emotions as the model is already validated, and emotions are still behavioral elements that could be interpreted differently. All of the 11 input variables were then considered for training and testing. Table 2 presents a summary of the statistics of all variables.

**Table 2:** Summary statistics of the dataset

	count	mean	std	min	25%	50%	75%	max
EAR	11,286	0.28	0.07	0	0.22	0.28	0.33	0.52
YAR	11,286	0.17	0.22	0	0.02	0.06	0.26	2.45
Rotation angle 1	11,286	-0.1	5.59	-26.66	-2.88	0.4	3.42	25.21
Rotation angle 2	11,286	0.48	3.99	-19.57	-0.83	0.59	2.51	28.21
Anger	11,286	0.64	2.34	-6.82	-1.16	0.48	2.54	8.39
Disgust	11,286	-6.75	2.36	-14.59	-8.24	-6.94	-5.6	5.28
Fear	11,286	-0.98	2.95	-11.34	-2.53	-0.72	0.6	11.68
Happiness	11,286	0.32	2.51	-7.08	-1.38	0.02	1.69	14.15
Sadness	11,286	6.27	3.19	-6.04	3.95	6.11	8.79	17.3
Surprise	11,286	-4.82	2.55	-11.85	-6.66	-5.03	-3.24	4.56
Neutrality	11,286	5.49	2.58	-4	4.02	5.32	6.83	18.81
Output	11,286	3.74	1.53	0	3	5	5	5

**Fig. 4:**
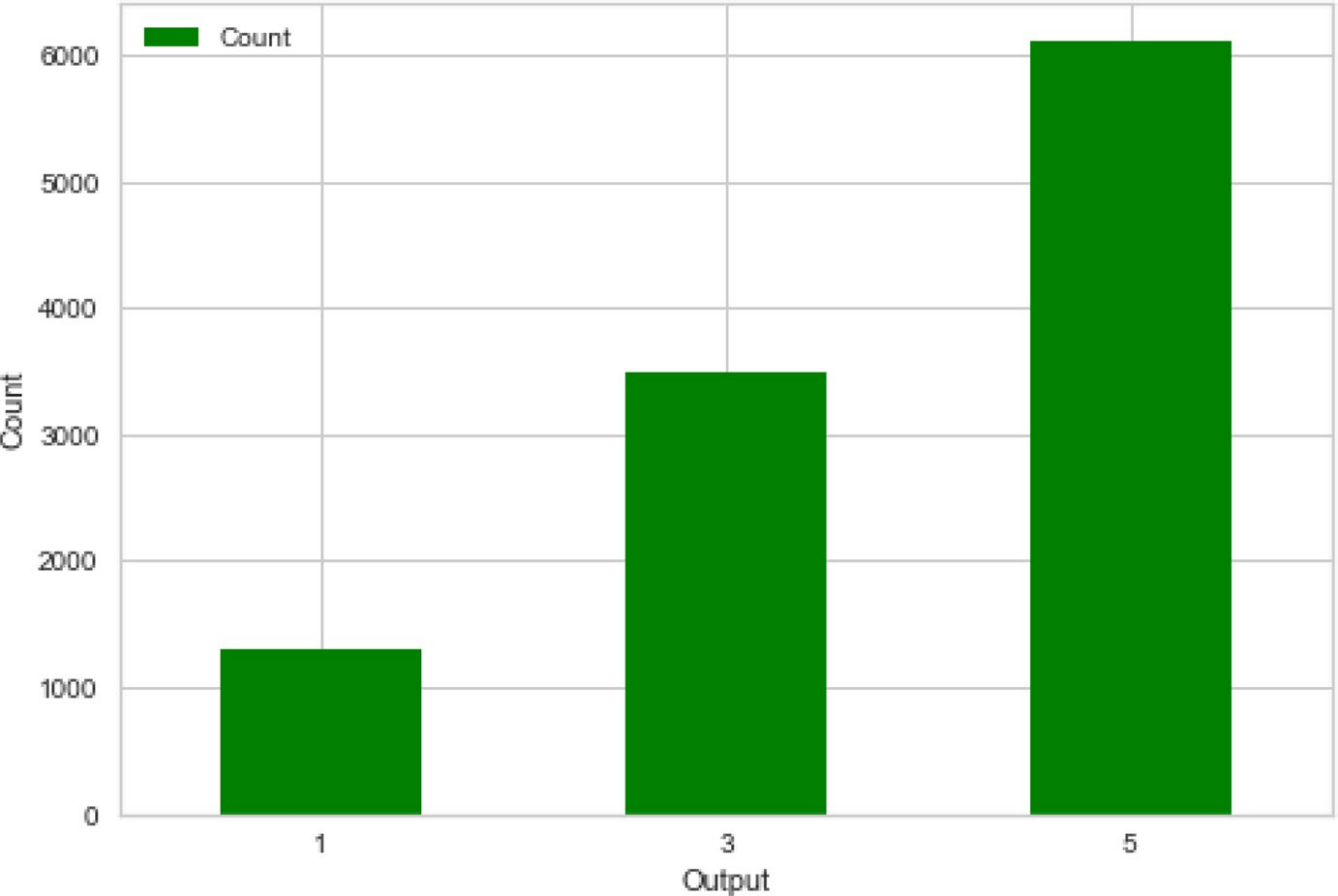
Output values for the final dataset

The final dataset contains 10,899 samples. The dataset had 1,294 frames (12%) with an output of 1 (not attentive). The dataset had 3,499 frames (32%) with an output of 3 (moderately attentive). The dataset had 6,106 frames (56%) with an output of 5(extremely attentive). Figure 4 shows the count of the different outputs of the final dataset. The unbalanced dataset is a result of 2 main factors. Firstly, the face detection algorithm does not detect the student's face while moving. Subsequently, these data points are not detected, usually related to less attentiveness while the student is moving. Secondly, the students were aware of the experiment; hence they were more focused than if they were in a standard e-learning class and not being recorded. To balance the unbalanced data, we have oversampled the data using SMOTE (Chawla et al., 2002). The models will then be trained and tested with the unbalanced and balanced data.

## Results and discussion 

### Drowsiness detector results

**Fig. 5:**
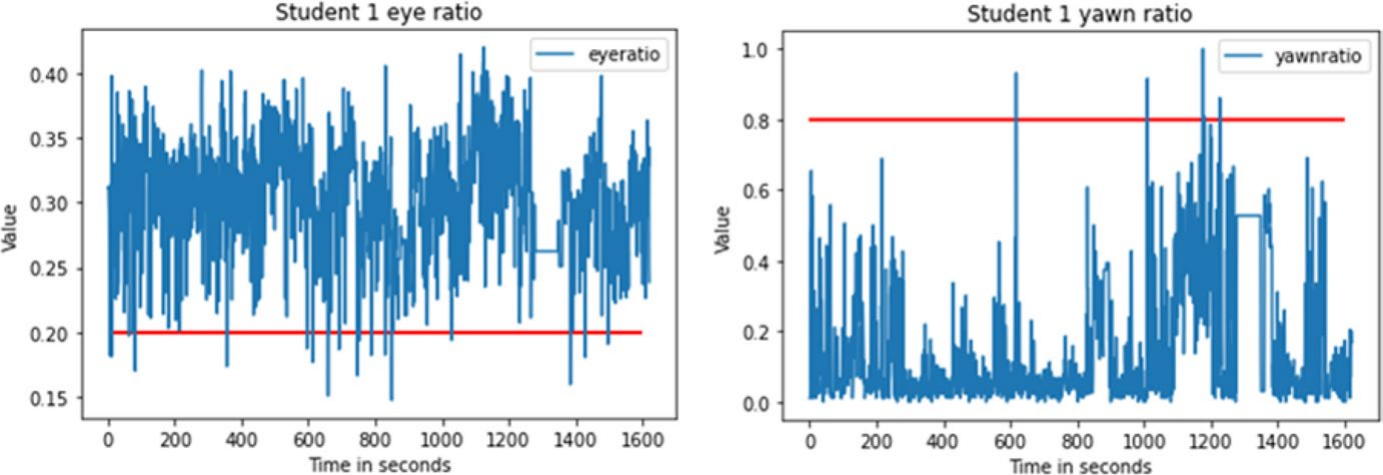
Eye and Yawn ratio for Student Student 1

The drowsiness detector has resulted in two variables for each student. Following (Shah et al., 2021), we used a threshold of 0.20 for detecting closed eyes. A threshold of 0.80 was used to detect when the student opened his mouth until the yawning level (Shah et al., 2021). Figure 5 presents the EAR and YAR for student student 1 as an example. The obtained EAR for all students shows that around 15% of the frames indicated students with eyes almost closed. As for the YAR, around 2.5% of the frames indicated students yawning.

### Head pose detector results

**Fig. 6:**
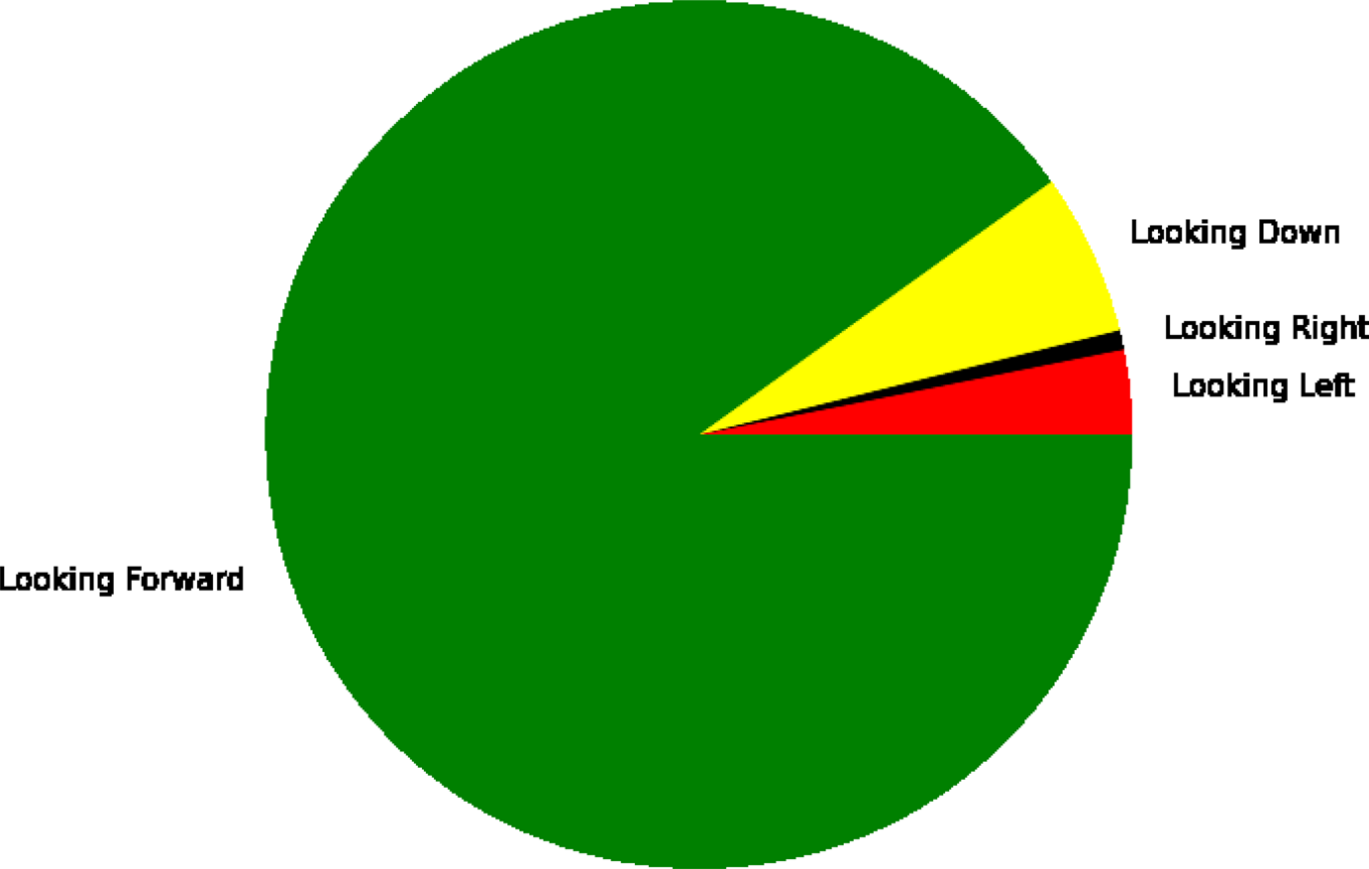
Head position for students from the *x* and *y* components

The head pose detector has generated two angles: angle 1 represents the *x* component of the head position, while angle 2 represents the *y* component. Figure 6 shows the results for the head pose data of the seven students, where the highest percentage refers to students looking forward, followed by looking down.

### Emotions detector results

**Fig. 7:**
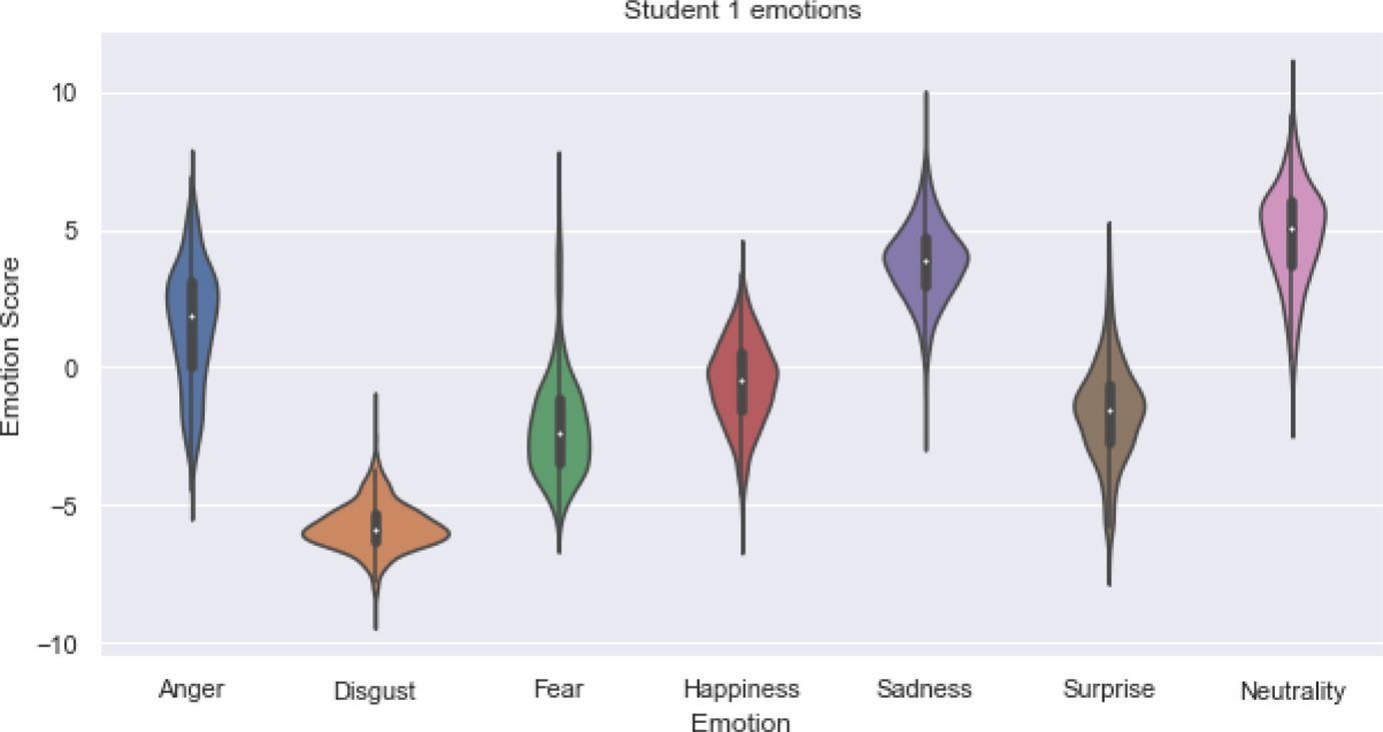
Emotions output for student 1

As for the Emotion detector, we applied the model proposed by (Khaireddin & Chen, 2021). When the face is detected for each frame, the model will represent the frame into the seven emotions. Figure 7 shows the emotions of student Student 1 throughout the watched lecture. The higher the emotion score, the higher the prediction of the emotion. The highest recorded emotions in all students are sadness and neutrality. This confirms (Tabassum et al., 2020), where the authors illustrated that sadness might indicate attentiveness because of the similarities between appearing calm and feeling sad.

Predicting* modeling* results.

After data exploration, we started the modeling phase by applying decision trees, random forest, XGBoost, and SVM.

Since the dataset is unbalanced, in this study, the AUROC OVR is used as it is sensitive to class unbalance, where we wanted to see the effect of using and not using oversampling in the different models.

Focusing on the accuracy, one can see that XGBoost and random forest achieved the best results with more than 80% in cross-validation, without and with oversampling, respectively. Both XGBoost and random forest can generate robust models that can be used. One can also notice that SVM can also generate a good model as its accuracy achieved more than 75% with cross-validation without oversampling. While focusing on the AUROC OVR, XGBoost and random forest have achieved more than 90%, strengthening the use of these models as per the accuracy scores mentioned earlier. SVM has also shown good AUROC OVR results, with more than 88% without oversampling. Decision trees are the poorest performers, where an accuracy of 70.51% and 71.83% were achieved with and without oversampling, respectively.

Table 3 summarizes the average scores for all of the models. A random search is performed to determine the best hyperparameters. The best model is the XGBoost (hyperparameter: max depth = 13) without oversampling, with an average AUROC OVR of 92.12% and an accuracy of 80.52%. The second-best model is the Random Forest with oversampling (hyperparameters: number of estimators = 300, max samples = 0.9, min samples split = 5, max depth = 22), with an average accuracy of 80.28% with AUROC OVR of 92.09%. The best model was a result of using the unbalanced dataset, hence AUROC OVR was used in addition to the accuracy to ensure that XGBoost is the best model to be used.

**Table 3:** Predictive models performance

	Accuracy (std)		AUROC OVR (std)	
	Unbalanced	SMOTE	Unbalanced	SMOTE
Decision Tree	71.83 (1.18)	70.51 (1.62)	81.85 (1.44)	77.03 (1.50)
Random Forest	79.94 (1.11)	**80.28 (1.20)**	92.01 (0.59)	**92.09 (0.65)**
XGBoost	**80.52 (0.99)**	79.71 (1.31)	**92.12 (0.57)**	91.40 (0.62)
SVM	77.11 (1.21)	75.00 (1.53)	88.79 (0.85)	87.87 (1.01)

### Follow up survey

Concerning the follow-up survey, the lecture average students' attentiveness was calculated, and the results show that the attentiveness was higher in the first half of the lecture. Although the students were aware of the experiment being recorded, still the second half had lower attention levels (Table 4). This result supports the proposal by (Lim, 2017) that student attentiveness drops after 10–15 min. Although the number of responses is low (N = 7), these results would help clarify students' behavior in online classes. For the first item, the students agreed with an average score of 4 that they maintain an attentive posture in online classes. As for the other three items, the average ranged from 3 to 3.5. Hence we can infer that posture can really help in assessing the attentiveness of students.

**Table 4:** Follow up survey adapted attending behavior results

Item	Mean	Std
In an online class, I maintain an attentive posture while people are speaking	4	0.462
In an online class, I give persons my complete attention when they are speaking	3	0.755
In an online class, I maintain eye contact with persons while they are speaking	3.5	0.744
In an online class, I respond non-verbally to let persons know I am listening	3.5	1.187

To summarize, results obtained by the considered ML algorithms show that XGBoost performs best in accuracy and AUROC OVR without oversampling. On the other hand, the second best results are for Random Forest regarding the accuracy and AUROC OVR while using SMOTE oversampling.

The analysis supports the initial hypothesis that using a model with emotional and non-emotional features would have an advantage in accuracy. The study benefited from using non-emotional measures on top of the emotional models. This study attained an accuracy of 0.852. The model accuracy of this study surpassed the accuracy achieved in (Zaletelj & Košir, 2017), where an accuracy of 0.753 was attained. Although it is difficult to directly compare the results due to differences in tools, datasets, experiment settings, and annotation methods, our results are comparable in terms of accuracy.

## Conclusions, limitations, and future work 

E-learning will continue to grow as more universities see the value of educating the masses remotely (Matzavela & Alepis, 2021; Mellieon & Robinson, 2021). Hence, understanding and reconceptualizing the fundamentals of teaching and learning is an important aspect nowadays (Matzavela & Alepis, 2021). In this study, we used ML to predict students' attentiveness in an e-learning setting. Every ML model was estimated using videos from students recorded in their favorable environment, usually their home. We used the videos to extract 11 features generated from the drowsiness, head pose, and emotions detector. The drowsiness and head pose detectors used OpenCV, while the emotions detector used a developed VGGnet. The dependent variable is the attentiveness of the student at a given moment. Subsequently, the estimated models can act as an effective and general tool for analyzing e-learning lectures. The model can generate an average attentiveness report for a given lecture, which would help educators design and enhance their lectures.

The results demonstrate the use of emotional and non-emotional measures in developing an ML model for predicting student attentiveness. We posit that implementing a combined ML model to detect student attentiveness could get an acceptable accuracy, where our best model using XGBoost reached an accuracy of 80.52% and an AUROC OVR of 92.12%. Using only one sensor (the webcam) helps facilitate learning analytics even when the students are not on campus.

The study has several limitations, which include, first, the ground truth data of human annotation is not entirely reliable and depends on the observer (Zaletelj & Košir, 2017). Second, the size of the training is still limited to a total of 7 student videos. Finally, the students were not wholly relaxed while recording the video; hence their recordings were affected.

For future work, we would recommend getting data from more students to help get more data points in developing the model. We also recommend starting the experiment with a game that would help the students be more relaxed and not focused on the recording. Finally, we recommend adding a variable showing the student's posture as per the outputs of the follow-up survey. This new variable needs to be tested with other variables to see if it would influence the model's accuracy. We also would like to study gamification on this context and understand its impact on student's attentiveness.

## Data Availability

The datasets generated during and/or analysed during the current study are not publicly available due to reasons of sensitivity but might be available from the corresponding author on reasonable request.
